# Age and educational level effects on the performance of normal elderly on category verbal fluency tasks

**DOI:** 10.1590/S1980-57642009DN30100010

**Published:** 2009

**Authors:** Helenice Charchat Fichman, Conceição Santos Fernandes, Ricardo Nitrini, Roberto Alves Lourenço, Emylucy Martins de Paiva Paradela, Maria Teresa Carthery-Goulart, Paulo Caramelli

**Affiliations:** 1 Departamento de Psicologia, Pontíficia Universidade Católica do Rio de Janeiro, Rio de Janeiro, RJ, Brazil; Departamento de Neurologia, Universidade de São Paulo, São Paulo, SP, Brazil.; 2 Laboratório de Pesquisa em Envelhecimento Humano - GeronLab, Universidade do Estado do Rio de Janeiro, Rio de Janeiro, RJ, Brazil.; 3 Departamento de Neurologia, Universidade de São Paulo, São Paulo, SP, Brazil.; 4 Grupo de Pesquisa em Neurologia Cognitiva e do Comportamento, Departamento de Clínica Médica da Faculdade de Medicina da Universidade Federal de Minas Gerais, Belo Horizonte, MG, Brazil.; 5 Departamento de Medicina Interna, Faculdade de Ciências Médicas, Universidade do Estado do Rio de Janeiro, Rio de Janeiro, RJ, Brazil.

**Keywords:** aged, animal fluency, educational status

## Abstract

Cognitive decline, particularly executive dysfunction, is observed in normal aging. In Brazil, the elderly population presents broad educational diversity. Category verbal fluency tests are frequently used to detect cognitive impairment, assessing executive function, language and semantic memory.

**Objective:**

To investigate the effects of age and education on category animal fluency task (CAF) in healthy elderly.

**Methods:**

We evaluated 319 healthy elderly from outpatient care units of two university reference centers of Rio de Janeiro and São Paulo. The sample was divided into two age, and five schooling subgroups. To be included participants had to demonstrate preservation of global cognitive functioning, independence for activities of daily living and not fulfill diagnostic criteria for dementia. All participants were submitted to neurological and neuropsychological evaluations.

**Results:**

There was a correlation between age and CAF performance (r= –0.26, *p*<0.01), which was not confirmed when years of education were included as a covariant in univariate ANCOVA. Significant differences were found in CAF performance among the different educational level groups on correlation analysis (r=0.42, *p*<0.01) and ANCOVA analysis (F=18.8, *p*<0.05). Illiteracy was associated with worst CAF performance, while university level was associated with best performance.

**Conclusion:**

The best CAF performance was found in the first years of schooling (literacy learning process) compared to illiteracy, and when finishing high school and starting university courses compared to all other educational levels. These stages are associated with significant gains in semantic memory and executive function which are critical for verbal fluency performance.

## Abstract (pt)

Declínio cognitivo, particularmente o funcionamento executivo é observado com aumento da idade em idosos saudáveis. No Brasil, a população idosa apresenta grande diversidade educacional. A fluência verbal semântica é um teste freqüentemente usado para detectar comprometimento cognitivo, avaliando funções executivas, linguagem e memória semântica.

**Objetivo:**

Investigar o efeito da idade e níveis educacionais na fluência semântica de animais em idosos saudáveis.

**Métodos:**

A amostra foi composta de 319 idosos saudáveis, divididos em duas categorias de idade e cinco níveis de escolaridade, provenientes de centros universitários de referência do Rio de Janeiro e São Paulo. Todos apresentaram: preservação do funcionamento cognitivo global, independência nas atividades da vida diária e não preencher critérios para demência. Todos os participantes foram submetidos à avaliação neurológica e neuropsicológica.

**Resultados:**

Houve correlação entre idade e desempenho na tarefa de fluência verbal animais (r= –0,26, *p*<0,01), não confirmada quando os anos de escolaridade foram incluídos como covariante no ANCOVA univariado. Diferenças significativas foram encontradas entre escolaridade e desempenho da fluência verbal animais tanto na análise de correlação (r=0,42, *p*<0,01) quanto na análise ANCOVA (F=18,8, *p*<0,05). Analfabetismo foi associado ao pior desempenho, enquanto o nível universitário foi associado com o melhor desempenho na fluência verbal de animais.

**Conclusão:**

A maior diferença de desempenho na fluência verbal animais foi encontrada nos primeiros anos de escolarização (processo de aprendizagem da alfabetização) quando comparado com os analfabetos e quando se finaliza o ensino médio e ingressa na universidade comparado aos demais níveis de escolaridade. Estes estágios estão associados com o aperfeiçoamento da memória semântica e de funções executivas, que são essenciais para o desempenho no teste de fluência verbal.

The elderly population has grown significantly in the last decade in developed countries such as Brazil,^1,2^ set to rank sixth in number of aged individuals by 2025.^3^ The normal aging process can lead to cognitive decline, including impairment of executive functions.^4-6^ A hypothesis for this decline is the physiological changes in frontal lobe structure and function.^7^ In addition to the fact that Brazil is a country with increased life expectancy, a marked heterogeneity of educational level also occurs, with part of the population having little or no schooling.^8^

Category verbal fluency^9^ (CVF) is a very useful and sensitive test for cognitive deterioration,^10-16^ assessing executive functions (mental organization, strategies for search), semantic and working memory, speed processing and language (size of vocabulary).^17,18^

Several investigators have reported high diagnostic accuracy provided by CVF tests in differentiating frontotemporal dementia, Alzheimer’s disease (AD) and Parkinson’s disease.^19,20^ Moreover, many studies have reported that CVF tasks yield high sensitivity in discriminating the early stages of dementia from normal aging.^21-23^

A review by Schwartz et al.^24^ demonstrated that poor performance on CVF is usually related to temporal lobe pathology, which can be corroborated by the fact that AD patients present poor performance on CVF.^11,14^

Age and education also can affect CVF, besides their clear influence on general cognition. Previous reports have pointed to the importance of educational level or literacy in cognitive function performance.^25-27^ Previous studies have also found effects of these variables on CVF.^13,10, 28^.

Brucki and Rocha in 2004^10^ observed that educational level significantly influenced the performance of healthy elderly in Category Animal Verbal Fluency (CAF), but the same did not occur with age. These same authors noted that this difference became even more apparent when comparing groups with large differences in education, or individuals with low education, such as illiterates, against those with greater than or equal to eight years of schooling. Mathuranath et al.,^13^ in their study in an Indian population, have shown that the influence of education could be explained by the impact of education on linguistic skills. This study showed performance below the average found in the literature.

Therefore, Brazilian and international studies reveal the necessity of exploring the effects of age and especially education on CVF performance. The goals of the present study were to investigate the effects of age and schooling on the CAF task in elderly without cognitive impairment and to discuss the different systems and cognitive functions involved in normal aging.

## Methods

### Subjects

The sample consisted of 319 (203 women and 116 men) cognitively healthy elderly who received outpatient care in university reference centers from Rio de Janeiro and São Paulo. They included 270 subjects from the Behavioral and Cognitive Neurology Unit of the University of São Paulo School of Medicine and 49 individuals followed at the Human Aging Research Laboratory – GeronLab from the University of the State of Rio de Janeiro. They were divided into two age groups: 203 aged less than or equal to 75 years (Youngest-old – Y) and 116 aged older than 75 years (Oldest-old – O). Subjects were also divided into five groups according to educational level: 0 years; 1–4 years; 5–8 years; 9–11 years and 12 or more years. All participants were submitted to physical and neurological examination and to global cognitive evaluation with the Mini-Mental State Examination (MMSE).

The study was approved by the Ethics Committee from both university centers and all participants signed the written informed consent.

In order to be included in the study, participants had to fulfill the following criteria:

1) preservation of global cognitive functioning documented by performance above education-adjusted scores on the MMSE;^29,30^
2) independence in activities of daily living documented by the Pfeffer Functional Activities Questionnaire^31^ or the Lawton scale;^32^
3) do not meet DSM-IV^33^ criteria for dementia.

The participants’ demographic and clinical characteristics are shown in Table 1.

**Table 1:** Demographic and clinical information.

	N	Mean	SD	Minimum	Maximum
MMSE	319	25.57	3.32	14.00	30.00
Age	319	72.87	7.08	60.00	92.00
Education (years)	319	6.84	5.50	0.0	17.00
Total Word generation VAF	319	14.37	4.39	4.00	28.00

MMSE, Mini-mental state examination; SD, standard deviation; VAF, verbal animal fluency.

### Instrument

Participants were instructed during the CAF task to generate as many animal names as possible during a 1 minute limit time. The following instructions were given:** “Now, you will say animals’ names, as many as you can. It can be any kind of animal, whose name begins with any letter. Just try to say it as quickly as you can”**. The participants were asked if they had understood. If instructions remained unclear or the participant made a mistake demonstrating they had not understood the instruction (i.e. begin with a category other than animals such as supermarket items), the test was stopped and the instructions were repeated. During the tasks, the examiner encouraged the participants to try to think of more words.

The following items were considered errors: intrusions (i.e.: when appropriate answers for a letter or category were given, but inappropriate in terms of category used at the time); perseverations (i.e. same words were repeated twice or more); general category error (when the participant stated a general category while on specific items; e.g.: fish and shark; fish is error and shark is correct). Examples of acceptable and unacceptable answers are shown in Table 2.

**Table 2:** Examples of answers given by the same subject.

Acceptable answers	Unacceptable answers
1 - dog	2 - poodle
3 - salmon	4 - fish (general category error)
5 - dinosaur	6 - dragon (mystic animal)
7 - monkey	8 - primate (general category error)
9 - bear	10 - papaya (intrusions)
11 - cat	12 - bear (perseveration)

### Statistical analysis

Descriptive analyses of the sample’s age, education, gender, MMSE scores were performed.

Pearson’s correlation between age and education was performed. Univariate analysis (t test and ANOVA) was conducted comparing education across the age groups and comparing age in years between educational level groups.

Pearson’s correlation’s was also conducted between age in years and total number of correct animal names. The age effect was further explored through analysis of covariance (ANCOVA), including education in years as a covariant.

In order to investigate the effects of education, Pearson’s correlation was conducted, testing the association between years of education and total number of correct animal names. To confirm these results, an ANCOVA test including age in years as a covariant followed by Least Significant Difference (LSD) post hoc analysis was conducted.

All analyses were performed between educational and age groups and total numbers of words generated. The statistical software SPSS, version 12.0 was used. The significance level considered was *p*<0.05or *p*<0.01.

## Results

The results of the descriptive analysis are shown in Tables 3 and 4. There were significant differences in education between the age groups (t=4.97, *p*<0.01) and significant age difference between the groups of educational level (F=10.72, *p*<0.01). Age and education in years were negatively correlated, according to Pearson’s analysis (r= –0.339, *p*<0.01).

**Table 3:** Mean and Standard Deviation of education (years) by age group.

Age group	Education (years)	Mean (SD)
Less/equal to 75 years	7.96 (5.66)	p<0.001
Greater than 75 years	4.89 (4.62)	

SD, standard deviation.

**Table 4:** Mean and standard deviation of age (years) in education level groups.

Education groups	Age (years)				
	N	Mean	(SD)	Range	
0	51	74.92	5.97	64-88	p<0.01
1-4	104	75.43	7.35	60-92	
5-8	52	72.09	7.09	60-85	
9-11	50	70.08	6.27	60-85	
≥12	62	69.75	6.00	60-83	

SD, standard deviation.

### Age effect

The relationship between different age ranges and task performance was compared through Pearson correlation. A significant and negative correlation was observed between age and CAF (r= –0.26, *p*<0.01). No significant age effect remained when education was included as a covariant in ANCOVA analysis (F=0.50, *p=*0.48). Mean and standard deviation of age groups are shown in Table 5.

**Table 5:** Mean and standard deviation of number of correct animal names on CAF in age and educational groups.

	Mean	SD
**Age groups**		
Less/equal to 75 years	14.9	4.6
Greater than 75 years	13.5	3.9
**Educational groups**		
0	11.5	3.6
1-4	13.6	3.6
5-8	14.3	3.9
9-11	15.4	4.6
≥12	17.2	4.8

### Educational effect

There was a significant and positive correlation between schooling and CAF (r=0.42, *p*<0.01). ANCOVA analysis showed a significant schooling effect on CAF performance (F=12.1, *p*<0.01). Mean and standard deviation of educational groups are depicted in Table 4. LSD Post hoc analysis showed that illiterate (0 years) groups had worst CAF performance than any other schooling group (*p*<0.01), while university level (12 or more years) had better performance when compared to the other educational level groups (*p*<0.01). Groups 1–4 years, 5–8 years and 9–11 years did not differ among each other (*p>*0.05). Mean and standard deviation of number of correct animal names in educational groups are show in Table 5. Table 6 presents the interaction between age and education during CAF.

**Table 6:** Interaction between aging and education levels in CAF performance.

Education groups	£ 75years' old				> 75 years' old		
	Mean	SD	Cutoff		Mean	SD	Cutoff
0 years	11.43	3.94	3.55		11.56	3.32	4.92
1-4 years	14.02	3.69	6.64		13.24	3.59	6.06
5-8 years	14.59	4.10	6.39		13.66	3.81	6.04
9-11 years	15.59	4.30	6.99		14.82	3.73	7.36
≥12 years	17.21	4.98	7.25		17.36	4.15	9.06

SD, standard deviation; Cutoff < -2.0 SDs from mean, abnormal performance.

## Discussion

In the present study, the effects of age and education on the performance of cognitively healthy elderly subjects on the CAF were investigated. The analysis of age effects indicated that the total number of correct animal names produced was negatively associated with years of age.

The variable age was strongly associated with years of schooling. The frequency of low education was higher in the oldest group (>75 years’ old), while high education level predominated in the youngest group (≤75 years’ old). In this sense, when the sample was divided into two age groups, a significant difference in years of education between groups emerged. Isolating the interaction of education and aging, the CAF performance did not differ between the age groups. These findings reveal that besides the association of aging on category verbal fluency task, the interaction with education is an important factor to be controlled.

Similar results were found by national and international researchers,^10,13^ namely a marked association between years of education and animal names produced in verbal fluency tasks. The diversity of educational levels in the sample allowed them to be split into five subgroups, ranging from illiterate to university degree level. The results, including age as a covariate, clearly showed that the illiterate group had the worst performance on CAF, while the university group (≥12 years) presented the best scores compared to other educational levels.

This observation points to the importance of literacy for cognitive development and to the conclusion of high school as a second marker for the refinement of cognitive abilities. The influence of illiteracy and high levels of education on CVF performance has also been recognized previously by some of the authors.^11^ Other findings supporting the importance of schooling on CVF is that low education constitutes a probable risk factor for cognitive impairment, even in elderly without dementia.^25,26,34^

One of the cognitive domains required for good verbal fluency performance is the executive functions, especially in terms of flexibility and initiative abilities. It is well recognized that executive functioning skills are also correlated with education, particularly reading ability, since verbal strategies used to search information are achieved through the literacy process.^35^ Moreover, reading and writing could lead directly to enrichment of neural networks.^36^ Thus, literacy and high education would provide verbal strategies, improve executive abilities and, consequently, verbal fluency performance. It is known that CVF tasks such as animals per minute, require activation of the semantic memory system, but since many sub-categories within the “animal” field also exist, more flexibility is required, thus making the test also dependent on executive functioning.

In conclusion, the number of animal names produced on the CVF task was higher in healthy elderly aged 75 years or less and with high levels of education. The most significant improvement in scores on the CVF was found in the first years of schooling (literacy learning process) compared to illiterate individuals, and when finishing high school and enrolling at university compared to all other education levels. These schooling abilities are particularly involved in semantic memory and executive function performance, cognitive processes required during verbal fluency paradigms. As CVF is an important tool for detecting semantic and executive function impairment in healthy elderly, this study revealed the importance of taking into account the schooling variable in countries like Brazil, which have a broad cultural diversity. Further studies comparing the verbal fluency performance of patients with dementia, mild cognitive impairment and healthy elderly subjects are needed to gain a better understanding of the interaction between poor cognitive performance as a result of education and age, and the effects of pathological processes.
